# Moving beyond the “Five Freedoms” by Updating the “Five Provisions” and Introducing Aligned “Animal Welfare Aims”

**DOI:** 10.3390/ani6100059

**Published:** 2016-09-23

**Authors:** David J. Mellor

**Affiliations:** Animal Welfare Science and Bioethics Centre, Institute of Veterinary, Animal and Biomedical Sciences, Massey University, Palmerston North 4442, New Zealand; D.J.Mellor@massey.ac.nz; Tel.: +64-21-390-855

**Keywords:** European Welfare Quality assessment system (WQ^®^), Five Animal Welfare Aims, Five Domains Model, Five Freedoms, Five Provisions, negative and positive experiences and states

## Abstract

**Simple Summary:**

A Five Provisions/Welfare Aims paradigm has been formulated as a coherent alternative to the Five Freedoms. It retains the memorable simplicity of the original paradigm and is linked to it, but avoids the acknowledged complications that arise by using the term “freedoms”. Also, it accommodates current scientific understanding of animal welfare, is easily understood and provides guidance on beneficial objectives for animal welfare management. It is an evocative and engaging paradigm anticipated to be of particular interest to non-specialist members of the lay public who are concerned about animal welfare.

**Abstract:**

Although the Five Freedoms paradigm has been very influential in shaping animal welfare thinking for the last two decades, it has two key disadvantages. First, the focus on “freedom” from a range of negative experiences and states has been misunderstood in a number of quarters to mean that complete freedom from these experiences and states is possible, when in fact the best that can be achieved is for them to be minimised. Second, the major focus of the Freedoms on negative experiences and states is now seen to be a disadvantage in view of current understanding that animal welfare management should also include the promotion of positive experiences and states. The challenge therefore was to formulate a paradigm that overcame these two main problems and yet was straightforward enough to be accessible to non-specialists, including members of the lay public who are interested in animal welfare. This was achieved by highlighting the Five Provisions, originally aligned with the Five Freedoms, but now updated to direct welfare management towards activities that both minimise negative experiences or states and promote positive experiences or states as specified by particular Animal Welfare Aims assigned to each Provision. Aspects of the four welfare principles from the European Welfare Quality assessment system (WQ^®^) and elements of all domains of the Five Domains Model for animal welfare assessment have been incorporated into the new Five Provisions/Welfare Aims paradigm. Thus, the paradigm is easily understood and provides clear guidance on beneficial objectives for animal welfare management. It is anticipated that the paradigm will have application to many species found in a wide range of circumstances.

## 1. Introduction

Two recent papers [1,2] considered the impact of the Five Freedoms paradigm on animal welfare thinking and management and acknowledged that the Freedoms have been profoundly influential since their formulation in 1993/1994 [3,4,5]. This was due in part to the memorable simplicity of the Freedoms as stated [2,5], but, as noted before [1], it was due especially to their alignment with Five Provisions which represented practical advice on how each Freedom might be achieved (Table 1). Perusal of many early and current codes of practice or welfare reveals the Provisions to have been incorporated as major foundational elements designed to improve animal welfare (e.g., [6,7,8,9,10,11]), but usually without naming them as Provisions [1]. Likewise, in the wider animal welfare literature (e.g., [12,13,14,15,16]), the Freedoms have been presented together with the aligned Provisions, but usually without explicit categorisation of the Provisions [1]. An example of a common format is: Freedom from thirst, hunger and malnutrition by providing ready access to fresh water and a diet to maintain full health and vigour (e.g., [2,17]).

Notwithstanding its widely beneficial influence, the Five Freedoms paradigm has two key disadvantages [1,2]. First, although originally intended to mean “as free as possible from” each of the experiences or states included in the Freedoms [4,5], it has often been apparent to the author, during extensive interactions, that some animal-based scientists not directly involved in animal welfare and some lay animal protectionists have come to regard the Freedoms, implicitly or explicitly, as “completely achievable” and even as “rights” [1]. To do so is biologically inaccurate and misleading [1,18]. This is because the negative experiences, for example, of thirst, hunger and pain are in fact required to elicit the drinking of water, the eating of food and the avoidance or withdrawal from injury-inducing events, and because all such behaviours are essential to secure the ongoing survival of the animal [19,20,21]. This is especially the case when the internal imbalances and/or challenging external conditions that give rise to these experiences are significant, and, at the extreme, are life-threatening. These survival-critical experiences, and other similarly aversive ones such as breathlessness, nausea and fear [18,22,23,24], can therefore never be eliminated, but, by appropriate application of the Provisions (Table 1), they can be reduced to tolerably low levels that nevertheless still elicit the required behaviours [1,18,21].

Second, when formulated, the Five Freedoms and aligned Provisions sought to focus attention on the negative experiences and states that were understood to contribute to welfare problems of serious concern at the time [3,4]. Thus, a major aim was to motivate the discovery and application of practical remedies for these problems. In short, the focus was on “problems” and the aim was to be “free” of them [1]. Moreover, this problem-solving approach has undoubtedly been very successful (e.g., [5,13,14,15,16,25,26,27,28,29,30]). However, the strong emphasis on “negatives” is now seen to be a limitation of the Five Freedoms paradigm [2], especially in light of the increasing recognition currently being given to positive experiences or states that animals may have and the ways those experience or states may be promoted [15,17,18,19,21,31,32,33,34,35].

Notwithstanding these limitations, John Webster [2] correctly pointed out that the Five Freedoms were not intended to represent an overall picture of the mental state and welfare status of animals, the details of which greatly interest scientists, veterinarians and other such animal welfare specialists. Rather, they were intended to be no more than a memorable set of signposts to guide appropriate action for non-specialists [2], such as the lay public including members of animal welfare NGOs. The role of addressing this audience remains as worthwhile today as it was when John Webster formulated the Five Freedoms paradigm in 1993/1994 [3,4]. Accordingly, the purposes of the present brief paper are to suggest a reconfiguration of the paradigm in a way that retains the memorable simplicity of its original formulation, retains a link to the original yet avoids complications arising by use of the term “freedoms”, and appropriately accommodates current scientific understanding of the foundations of what animal welfare represents.

## 2. Key Issues and Recommended Steps

Three key issues have been noted above: *first*, the Provisions, as opposed to the Freedoms, have probably been more influential in improving animal welfare by providing practical advice on the minimisation of negative experiences and states [1,2]; *second*, most of the negative experiences referred to in the Five Freedoms paradigm (Table 1), and other such experiences [18], can only be minimised, not eliminated, because when the internal or external conditions that give rise to them could eventually become life-threatening they are essential for motivating animals to engage in very specific behaviours that are critical for securing their survival [18,20]; and *third*, the earlier primary aim of animal welfare management, i.e., to minimise negative experiences and states, must now be broadened to *also* include the recognition and promotion of positive experience or states [1,21,35].

Three steps are therefore recommended; *first*, avoid reference to the Five Freedoms in order to reduce misconceptions and confusion; *second*, give greater prominence to the Five Provisions, appropriately updated so that they address both negative and positive experiences or states; and *third*, align each of the provisions with salient animal welfare aims that emphasise animals’ subjective experiences that most directly influence their welfare (Table 2).

The strong emphasis on subjective experiences, or affects, recognises, at least conceptually, that an animal’s welfare state depends on the net balance between the significant negative and positive experiences it may have at any one time [1,18]. It also recognises that “biological functioning” and the experience of “affective states” are now understood to be dynamically integrated elements within the body operating as a whole entity [18,36,37], so that the emphasis on affects in this updated paradigm incorporates both of these widely discussed approaches to understanding animal welfare [29,38,39].

## 3. Integrating Elements of other Conceptual Frameworks into this Updated Paradigm

The European Welfare Quality assessment system for farm livestock (WQ^®^), which specifically excludes utilisation of the Five Freedoms paradigm [40,41], enunciates four “welfare principles” of “good feeding”, “good housing”, “good health” and “appropriate behaviour” [40,42]. These principles focus attention on the practical measures needed to achieve desirable welfare outcomes where use of the words “good” and “appropriate” accommodate the dual aims of minimising negative experiences and promoting positive ones. Likewise, the Five Domains Model for animal welfare assessment developed in 1994 [43] and regularly updated since, most recently in 2015 [18], identifies “nutrition”, “environment”, “health” and “behaviour” as four physical/functional domains and thereby focuses attention on the practical management of animals. The Model also incorporates a fifth domain of “mental state” to represent animals’ welfare status, understood as the overall subjective (or affective) outcome of both negative and positive experiences generated by internal states or external circumstances captured by considering the first four domains [18]. Thus, the Five Domains Model also accommodates the dual aims of minimising negative experiences and promoting positive ones [1,18].

Clearly, both conceptual frameworks have relevance to updating the Five Provisions (Table 2). Thus, the four WQ^®^ principles have been adopted as identifiers for the first four Provisions, and are concordant with the first four physical/functional domains of the Five Domains Model. The fifth Provision, aligned with the fifth domain of the Model, refers to “positive mental experiences” and is intended to direct greater attention towards the promotion of positive welfare states in line with current thinking (e.g., [21,29,32,33,34,35]). Importantly, it is expressed in terms of providing animals with “opportunities” to have pleasurable experiences, as suggested elsewhere [17,18,19,21,34].

The Animal Welfare Aims of each Provision in effect reconfigure the Five Freedoms by more realistically referring to the minimisation of negative experiences or states, and now, by also explicitly including the promotion of salient positive experiences or states (Table 2). The examples of affects included in the Aims have been abbreviated from more extensive lists available elsewhere [18,32], which interested readers may consult. It is anticipated that these updated Provisions and aligned Animal Welfare Aims will have application to many species and circumstances in addition to livestock kept on farms.

Note, however, that in characterising animal welfare in terms which are more easily assimilated by lay people than is the far greater detail regarding internal states, external circumstances and the related affects embodied in the WQ^®^ system and the Five Domains Model used for the assessing animal welfare [1,18,40,42], the simplicity of the reconfigured Five Provisions/Welfare Aims paradigm outlined here necessarily constitutes a truncated description of what animal welfare is currently understood to represent. Nevertheless, the paradigm is fully consistent with that understanding [1].

## 4. Discussion

This reconfigured Five Provisions/Welfare Aims paradigm is future focused. It recognises that what is important practically is the Provisions and it provides targets for them in the form of aligned Animal Welfare Aims. Moreover, the Aims strongly emphasise what animals experience subjectively, i.e., their affects. This emphasis is intended to motivate those responsible for animal care to focus both on minimising the negative subjective experiences and on promoting the positive experiences animals may have. In other words, the overall objective is to provide opportunities for animals to “thrive”, not simply “survive” [1,44], by applying practical measures that do much more than merely meet their basic needs for water, food, shelter and disease reduction [1,7,17,18,19,29,32,33,45].

As noted before [1], opportunities to thrive include making available: variable environments with a congenial balance between predictability and unpredictability; access to preferred sites for resting, thermal comfort and voiding excrement; environmental choices that encourage exploratory and food acquisition behaviours which are enjoyable; a variety of feeds having pleasurable tastes, smells and textures; and circumstances that enable social species to engage in bonding and bond affirming activities and, as appropriate, other affiliative interactions such as maternal, paternal or group care of young, play behaviour and sexual activity [1,7,17,18,19,29,32,33,45]. The overall objective, as indicated in the Animal Welfare Aim No. 5 (Table 2), is to provide a range of opportunities for animals to experience comfort, pleasure, interest, confidence and a sense of control.

It would obviously be unrealistic practically and economically to aim to deliver all such opportunities immediately. However, in accord with the principle of incremental improvement [46], it is suggested that those responsible for animal care and management in all animal use sectors should utilise information that is now widely available (e.g., [1,14,18,31,32,33,47,48,49]) to guide the speedy implementation of whatever small, medium or large changes may be feasible in their particular circumstances [21,34,35].

## 5. Conclusions

The Five Provisions/Welfare Aims paradigm presented here retains the memorable simplicity of the original Five Freedoms formulation. It also retains a link to that original formulation, yet avoids complications arising from use of the term “freedoms”. Importantly, it accommodates current scientific understanding of the foundations of what animal welfare represents. Finally, it is easily understood and provides clear guidance on beneficial objectives for animal welfare management. It is therefore recommended as a coherent and engaging substitute for the Five Freedoms paradigm, and is anticipated to be of particular interest to non-specialist members of the lay public who are concerned about animal welfare.

## Figures and Tables

**Table 1:** The original Five Freedoms and Five Provisions for promoting farm animal welfare [3,4,5].

Freedoms	Provisions
1. Freedom from thirst, hunger and malnutrition	By providing ready access to fresh water and a diet to maintain full health and vigour
2. Freedom from discomfort and exposure	By providing an appropriate environment including shelter and a comfortable resting area
3. Freedom from pain, injury, and disease	By prevention or rapid diagnosis and treatment
4. Freedom from fear and distress	By ensuring conditions and treatment which avoid mental suffering
5. Freedom to express normal behaviour	By providing sufficient space, proper facilities and company of the animal’s own kind

**Table 2:** The updated Five Provisions and aligned Animal Welfare Aims.

Provisions ^1^	Animal Welfare Aims ^2^
1. *Good nutrition:* Provide ready access to fresh water and a diet to maintain full health and vigour	Minimise thirst and hunger and enable eating to be a pleasurable experience
2. *Good environment:* Provide shade/shelter or suitable housing, good air quality and comfortable resting areas	Minimise discomfort and exposure and promote thermal, physical and other comforts
3. *Good health:* Prevent or rapidly diagnose and treat disease and injury, and foster good muscle tone, posture and cardiorespiratory function	Minimise breathlessness, nausea, pain and other aversive experiences and promote the pleasures of robustness, vigour, strength and well co-ordinated physical activity
4. *Appropriate behaviour:* Provide sufficient space, proper facilities, congenial company and appropriately varied conditions	Minimise threats and unpleasant restrictions on behaviour and promote engagement in rewarding activities
5. *Positive mental experiences:* Provide safe, congenial and species-appropriate opportunities to have pleasurable experiences	Promote various forms of comfort, pleasure, interest, confidence and a sense of control

**^1^** The names of the Five Provisions (in italics) parallel those of the Five Domains Model for animal welfare assessment, and the names of numbers 1–4 are the same as the four European Welfare Quality (WQ^®^) principles; **^2^** Note that the first four Animal Welfare Aims refer both to minimising negative experiences or situations and to promoting positive ones, and the fifth one is entirely directed at promoting positive experiences. This is in keeping with the now accepted objective of giving greater attention to the promotion of positive welfare states.
